# A Quantile General Index Derived from the Maximum Entropy Principle

**DOI:** 10.3390/e24101431

**Published:** 2022-10-08

**Authors:** Tomonari Sei

**Affiliations:** Graduate School of Information Science and Technology, The University of Tokyo, 7-3-1 Hongo, Bunkyo-ku, Tokyo 113-8656, Japan; sei@mist.i.u-tokyo.ac.jp

**Keywords:** check loss function, general index, SDGs, unsupervised learning

## Abstract

We propose a linear separation method of multivariate quantitative data in such a way that the average of each variable in the positive group is larger than that of the negative group. Here, the coefficients of the separating hyperplane are restricted to be positive. Our method is derived from the maximum entropy principle. The composite score obtained as a result is called the quantile general index. The method is applied to the problem of determining the top 10 countries in the world based on the 17 scores of the Sustainable Development Goals (SDGs).

## 1. Introduction

Consider a data matrix, each row of which corresponds to a case, and each column represents a variable. Suppose that every variable has the meaning that a larger value indicates better. For example, ref. [1] investigated the efforts of countries to attain the SDGs (Sustainable Development Goals) and reported the 17 SDG scores for each country. The scores ranged from 0 to 100. In the report, a ranking of 163 countries on the basis of the average of the 17 scores was provided. We call such a procedure of ranking the simple sum method.

However, we sometimes find a paradoxical phenomenon in the simple sum method, in that a particular variable of a higher-score group is less than that of the remaining group. See Table 1 for illustration, where we separate the SDGs data into two groups: the 10 top countries on the basis of the simple sum method and the remaining 153 countries. The average values of each variable for the two groups are compared. On almost all the variables, the 10 top countries have larger averages than the remaining countries, as expected. However, there are reverse relations in the SDGs 12 and 13. The 10 top countries have an average value lower than the remaining countries on the two goals.

In this paper, we propose a linear weighting method that can avoid the reversal relation (in a random-decision sense). The higher-score group separated by the linear weight has average values greater than the remaining group with respect to all the variables. The idea behind the method is the objective general index (OGI; [2]), which is constructed to have a positive correlation with all the variables. The purpose of the OGI is the ranking and not the separation. The OGI is interpreted as a minimization problem of a free energy functional [3,4], which is the sum of the negative entropy and an internal energy functional. This interpretation also works in the current setting; see Section 2.

The problem of determining weights is unsupervised in the sense that no one knows the correct weights and classifications, which has been consistently discussed (e.g., [5,6]). There are many weighting methods for such purposes. Among them, the principal component analysis (PCA) is widely used. The PCA, however, does not always give positive weights; so, some modifications are necessary. It is known that a nonnegative version of the principal component analysis is a nonconvex and NP-hard optimization problem [7]. Another approach is the factor analysis, where a factor model refers to a set of multivariate distributions that have common latent factors (e.g., [8]). Although the factor analysis is quite flexible, it needs additional assumptions such as variance–covariance structures and often does not have a unique solution. In contrast, the quantile general index we propose is reduced to a convex optimization problem and is essentially unique as we will demonstrate. The Hirsch index (or h-index) is widely used for the evaluation of scientific research reports [9], and its further application has been recently investigated by [10]. We numerically compare our method with the h-index in Section 5.

The name of the quantile general index comes from the quantile regression developed by [11]. Indeed, the objective function we use is similar to those of the quantile regression; see the explicit form in Section 3. The essential difference here is that our problem is unsupervised, whereas the regression problems are supervised.

The general indices determine an ordering of the data. The problem of well ordering multivariate data was discussed by [12], where methods of ordering were classified into four categories: marginal ordering, reduced ordering, partial ordering, and conditional ordering. Our method is considered as marginal ordering on the weighted sum.

The paper is organized as follows. In Section 2, we define the quantile general index for continuous distributions and show that it is characterized by the maximum entropy principle. In Section 3, a finite-sample counterpart of the quantile general index is derived. In Section 4, a practical method that avoids the ambiguity of data lying on the separating hyperplane is proposed. We apply the method to the SDG data in Section 5, and we conclude in Section 6.

## 2. Quantile General Index for Continuous Distributions

The quantile general index for continuous probability distributions is defined first. The assumption of continuity avoids the difficulty caused by the non-smoothness of the objective function. The sample counterpart of the index is constructed in the subsequent section.

Suppose that we have a random vector $x={\left(x_{1},\ldots ,x_{d}\right)}^{⊤}$ following a probability distribution P on $R^{d}$, where ⊤ denotes the vector transpose. We assume that P has the probability density function p(x) so that $P\left(A\right)=\int_{A}p\left(x\right)dx$ for an event $A\subset R^{d}$. For given $h:R^{d}\to R$, we denote the expectation of a random variable h(x) by E[h(x)]=∫p(x)h(x)dx and the conditional expectation of h(x) given an event *A* by

$$E\left[h\left(x\right)|A\right]=\frac{\int_{A}p\left(x\right)h\left(x\right)dx}{\int_{A}p\left(x\right)dx}.$$

We deal with a class of general indices

(1) $$\begin{array}{c} g\left(x\right)=g\left(x;w,c\right)=\sum_{i=1}^{d}w_{i}x_{i}-c \end{array}$$

of x, where $w={\left(w_{1},\ldots ,w_{d}\right)}^{⊤}\in R_{+}^{d}$ and c∈R are called the weight vector and the threshold, respectively. Here $R_{+}$ denotes the set of positive numbers. The quantities w and *c* may depend on the underlying distribution P but do not depend on x itself.

For a given *g* of the form (1), the half spaces separated by the hyperplane g(x)=0 are denoted by

$$H_{g}^{+}=\left\{x|g\left(x\right)>0\right\}\;and\;H_{g}^{-}=\left\{x|g\left(x\right)<0\right\}.$$

The quantile general index is defined as follows.

**Definition** **1.** *A general index* $g\left(x\right)=\sum_{i}w_{i}x_{i}-c$ *is called the quantile general index of* x *if it satisfies the following two equations:*

(2) $$\begin{array}{c} P(H_{g}^{+})=\alpha \end{array}$$

*and*

(3) $$\begin{array}{c} E\left[w_{i}x_{i}\;|\;H_{g}^{+}\right]-E\left[w_{i}x_{i}\;|\;H_{g}^{-}\right]=1,\;i=1,\ldots ,d. \end{array}$$

*The weight* w *is calledthe optimal weight.*

Let us call $H_{g}^{+}$ and $H_{g}^{-}$ the positive and negative group, respectively. Equation (2) means that the fraction of the positive group is α. The threshold *c* is the upper α-quantile of the weighted sum $w^{⊤}x$ because $P(w^{⊤}x>c)=\alpha$ by (2). We call α *the acceptance ratio*. Equation (3) implies that the average of each variable $x_{i}$ on the positive group is greater than that on the negative group. Therefore, the reversal relation observed in Table 1 does not occur if we adopt the quantile general index.

**Figure 1:**
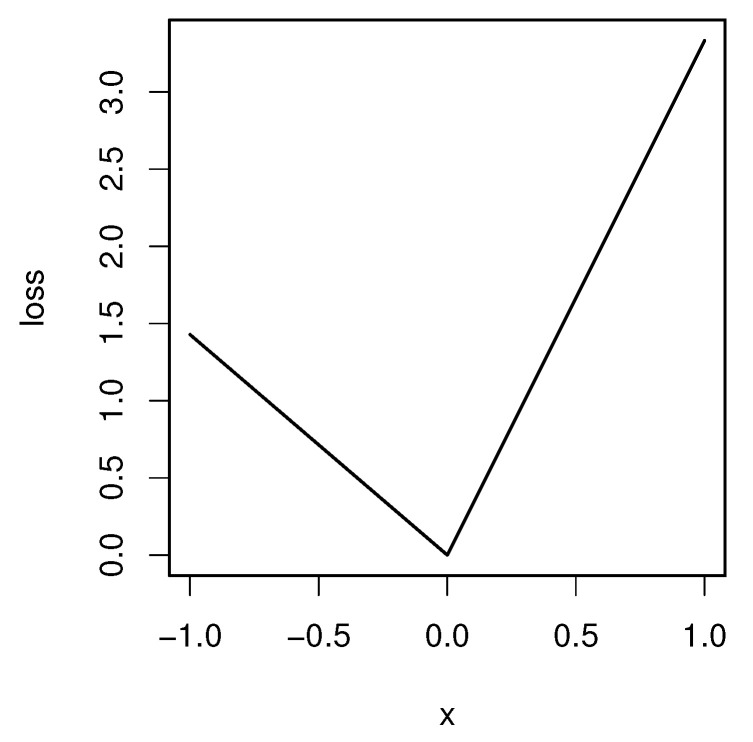
The check-loss function for α=0.3.

We now state the existence and uniqueness theorem of the quantile general index. For 0<α<1, we define the “check” loss function $\ell_{\alpha }:R\to R$ by

(4) $$\begin{array}{cc} \ell_{\alpha }\left(u\right)\; & =\frac{u^{-}}{1-\alpha }+\frac{u^{+}}{\alpha }, \end{array}$$

where $u^{+}=\max\left(u,0\right)$ and $u^{-}=\max\left(-u,0\right)$ are the positive and negative parts of *u*, respectively. See Figure 1 for the graph of $\ell_{\alpha }$. The function $\ell_{\alpha }$ is used in quantile regression [13]. The derivative of $\ell_{\alpha }\left(u\right)$ for u≠0 is

$$\begin{array}{cc} \ell_{\alpha }^{\prime }\left(u\right)\; & =-\frac{1}{1-\alpha }I_{\left\{u<0\right\}}+\frac{1}{\alpha }I_{\left\{u>0\right\}}, \end{array}$$

where $I_{\left\{u>0\right\}}$ is 1 if u>0 and 0 otherwise. The subgradient (e.g., [14]) at u=0 can be also defined but is not used here.

We define a convex function $F:R_{+}^{d}\times R\to R$ by

(5) $$\begin{array}{cc} F(w,c)\; & =-\sum_{i=1}^{d}\log w_{i}+E\left[\ell_{\alpha }\left(\sum_{j=1}^{d}w_{j}x_{j}-c\right)\right]. \end{array}$$

The main theorem is stated as follows.

**Theorem** **1.** *Let $x={\left(x_{1},\ldots ,x_{d}\right)}^{⊤}$ be a random vector with a probability density function on $R^{d}$ and assume that $E[x_{i}]$ exists for each i. Let 0<α<1. Then, the function F in (5) admits a minimizer $\left(w,c\right)\in R_{+}^{d}\times R$. The optimal w is unique, whereas c may not be unique. Furthermore, the general index $g\left(x\right)=\sum_{i}w_{i}x_{i}-c$ based on the minimizer (w,c) of F satisfies the conditions (2) and (3) of the quantile general index.*

**Proof.**  The proof of existence and uniqueness is given in Appendix A. We prove that the stationary condition of *F* is given by (2) and (3). The partial derivatives of *F* with respect to *c* and $w_{i}$ are

$$\begin{array}{cc} \frac{\partial F}{\partial c}\; & =-E[\ell_{\alpha }^{\prime }\left(g\left(x\right)\right)]\\ \; & =\frac{1}{1-\alpha }P\left(H_{g}^{-}\right)-\frac{1}{\alpha }P\left(H_{g}^{+}\right) \end{array}$$

and

$$\begin{array}{cc} \frac{\partial F}{\partial w_{i}}\; & =-\frac{1}{w_{i}}+E\left[x_{i}l_{\alpha }^{\prime }\left(g\left(x\right)\right)\right]\\ \; & =-\frac{1}{w_{i}}-\frac{1}{1-\alpha }E\left[x_{i}I_{\left\{g(x)<0\right\}}\right]+\frac{1}{\alpha }E\left[x_{i}I_{\left\{g(x)>0\right\}}\right]. \end{array}$$

Note that $P\left(H_{g}^{-}\right)=1-P\left(H_{g}^{+}\right)$, since P(g(x)=0)=0 from the assumption that x has a continuous distribution. Then, the equations ∂F/∂c=0 and $\partial F/\partial w_{i}=0$ (i=1,…,d) are equivalent to (2) and (3). □

**Example** **1.** *Let $x_{1}$ and $x_{2}$ be independent and identically distributed according to a continuous distribution. By the uniqueness of the optimal weight and symmetry, we have $w_{1}=w_{2}(=$w). We denote the upper α-quantile of $x_{1}+x_{2}$ by $y_{\alpha }$. Then, we have $c/w=y_{\alpha }$ from (2) and*

$$w=\frac{1}{E\left[x_{1}|x_{1}+x_{2}>y_{\alpha }\right]-E\left[x_{1}|x_{1}+x_{2}<y_{\alpha }\right]}$$

*from (3). For example, if $x_{i}$ has the standard normal distribution and α=1/2, then c=0, and $w=\sqrt{\pi }/2$.*

The quantile general index is derived from the maximum entropy principle in line with [4]. The entropy of a density function *p* is defined by

S(p)=−∫p(x)logp(x)dx.

Consider a class of transformations $T:R^{d}\to R^{d}$ of the form

$$T\left(x\right)=\left(w_{1}x_{1}-c_{1},\ldots ,w_{d}x_{d}-c_{d}\right),\;\left(w_{i},c_{i}\right)\in R_{+}\times R.$$

The push-forward density of *p* by *T* is defined by

$$\begin{array}{cc} \left(T_{♯}p\right)\left(x\right)\; & =p\left(T^{-1}\left(x\right)\right)\left|{\left(T^{-1}\right)}^{\prime }\left(x\right)\right|\\ \; & =p\left(\frac{x_{1}+c_{1}}{w_{1}},\ldots ,\frac{x_{d}+c_{d}}{w_{d}}\right)\frac{1}{w_{1}\cdots w_{d}}. \end{array}$$

This is the distribution of T(x) when the random variable x follows the distribution P. It is shown that the entropy of the push-forward density is

$$S\left(T_{♯}p\right)=S\left(p\right)+\sum_{i=1}^{d}\log w_{i}.$$

We also define an internal energy by

$$U\left(p\right)=\int p\left(x\right)\ell_{\alpha }\left(\sum_{i}x_{i}\right)dx,$$

where $\ell_{\alpha }$ is the check loss function in (4). The following theorem characterizes the quantile general index in terms of entropy. The proof is straightforward.

**Theorem** **2.** *The minimization problem of (5) is equivalent to*

$$\begin{array}{cc} \mathrm{Minimize} & \;\;U\left(T_{♯}p\right)-S\left(T_{♯}p\right)\\ \mathrm{subject}\;\mathrm{to} & \;\;T\left(x\right)=\left(w_{1}x_{1}-c_{1},\ldots ,w_{d}x_{d}-c_{d}\right),\\ \; & \;\;\left(w_{i},c_{i}\right),\ldots ,\left(w_{d},c_{d}\right)\in R_{+}\times R. \end{array}$$

*The threshold c in (5) is given by $c=\sum_{i=1}^{d}c_{i}$.*

## 3. Quantile General Index for Finite Samples

The quantile general index defined in the preceding section is valid only for continuous distributions. It is useful to define the index also for finite samples. Let $x_{\left(1\right)},\ldots ,x_{\left(n\right)}\in R^{d}$ be a sample of size *n*. We denote the *i*-th coordinate of $x_{\left(t\right)}$ by $x_{ti}$. We deal with a class of general indices $g_{t}=\sum_{i=1}^{d}w_{i}x_{ti}-c$, where $\left(w,c\right)\in R_{+}^{d}\times R$ may depend on the whole sample ${\left\{x_{\left(t\right)}\right\}}_{t=1}^{n}$ but does not depend on *t*.

The empirical counterpart of the objective function (5) is

(6) $$\begin{array}{c} F\left(w,c\right)=-\sum_{i=1}^{d}\log w_{i}+\frac{1}{n}\sum_{t=1}^{n}\ell_{\alpha }\left(\sum_{j=1}^{d}w_{j}x_{tj}-c\right) \end{array}$$

for $\left(w,c\right)\in R_{+}^{d}\times R$.

**Definition** **2.** *A general index $g_{t}=\sum_{i}w_{i}x_{ti}-c$ of $x_{\left(t\right)}$ for t=1,…,n is called the quantile general index if (w,c) minimizes the function (6).*

**Remark** **1.** *As described in Section 1, the objective function (6) is similar to that of the quantile regression defined by*

$$\frac{1}{n}\sum_{t=1}^{n}\ell_{\alpha }\left(y_{t}-\sum_{j=1}^{d}w_{j}x_{tj}\right),$$

*where $y_{t}$ is a response variable and $w_{1},\ldots ,w_{d}$ are regression coefficients. See [13] for a comprehensive study of the quantile regression.*

The following theorem is proved in a similar way to Theorem 1. See Appendix A.

**Theorem** **3.** *Suppose that there is no hyperplane of $R^{d}$ that contains all $x_{\left(t\right)}$. Then, the objective function F in (6) admits a minimizer (w,c). The weight vector w is unique. The threshold c is unique if nα is not an integer.*

Each case $x_{\left(t\right)}$ is classified into positive and negative groups according to $g_{t}>0$ and $g_{t}<0$, respectively. If the case $g_{t}=0$ does not exist, then the fraction of the positive (resp. negative) group is α (resp. 1−α), and the conditional expectation of $x_{ti}$ on the positive group is greater than that on the negative group. This is the desired dominance relation.

However, it is not always possible to classify the data into positive and negative groups, because $g_{t}$ may become 0 in some cases. Furthermore, the minimization of F(w,c) is not straightforward, since the function is not differentiable. In order to avoid these issues, we modify the method in Section 4.

For illustration, we calculate the quantile general index for the following examples.

**Example** **2.** *Consider the bivariate data*

x(1)=22,x(2)=21,x(3)=02,x(4)=00

*of sample size 4. Let the acceptance ratio be α=1/2. In this data, any set of three points is not on a straight line. Therefore, there exists the quantile general index by Theorem 3. We show that the solution is $w_{1}=2/3$, $w_{2}=4/3$, and c=8/3. We consider three disjoint subsets of $R_{+}^{2}$:*

$$A=\left\{w|w_{2}<2w_{1}\right\},\;B=\left\{w|w_{2}>2w_{1}\right\},\;C=\left\{w|w_{2}=2w_{1}\right\}.$$

*Let w∈A. Then, we have*

$$w^{⊤}x_{\left(1\right)}>w^{⊤}x_{\left(2\right)}>w^{⊤}x_{\left(3\right)}>w^{⊤}x_{\left(4\right)}$$

*Hence, the optimal c is between $w^{⊤}x_{\left(2\right)}$ and $w^{⊤}x_{\left(3\right)}$, since c is the upper 1/2-quantile of $\left\{w^{⊤}x_{\left(t\right)}\right\}$. For such c, the objective function (6) becomes*

$$\begin{array}{cc} F(w_{1},w_{2},c)\; & =-\log w_{1}-\log w_{2}+\frac{1}{4}\left(\frac{w^{⊤}x_{\left(1\right)}}{1/2}+\frac{w^{⊤}x_{\left(2\right)}}{1/2}-\frac{w^{⊤}x_{\left(3\right)}}{1/2}-\frac{w^{⊤}x_{\left(4\right)}}{1/2}\right)\\ \; & =\left(-\log w_{1}+2w_{1}\right)+\left(-\log w_{2}+w_{2}/2\right). \end{array}$$

*If F is minimized at some w∈A, then it must be $w_{1}=1/2$ and $w_{2}=2$ by the stationary condition, but this point does not belong to A. Hence, the optimal point does not exist in A.*

*If w∈B, then we have*

$$w^{⊤}x_{\left(1\right)}>w^{⊤}x_{\left(3\right)}>w^{⊤}x_{\left(2\right)}>w^{⊤}x_{\left(4\right)}$$

*and the objective function is*

$$\begin{array}{cc} F(w_{1},w_{2},c)\; & =\left(-\log w_{1}\right)+\left(-\log w_{2}+3w_{2}/2\right), \end{array}$$

*where $w^{⊤}x_{\left(2\right)}\leq c\leq w^{⊤}x_{\left(3\right)}$. It is shown again that the optimal point does not exist in B.*

*Therefore, the optimal point should be located in C, the boundary of A and B. The objective function is*

$$\begin{array}{cc} F(w_{1},2w_{1},c)\; & =-\log2-2\log w_{1}+3w_{1}, \end{array}$$

*where $c=w^{⊤}x_{\left(2\right)}=w^{⊤}x_{\left(3\right)}=4w_{1}$. The optimal solution is $w_{1}=2/3$, $w_{2}=4/3$, and c=8/3. The quantile general index is given by*

$$\left(\begin{array}{c} g_{1}\\ g_{2}\\ g_{3}\\ g_{4} \end{array}\right)=\left(\begin{array}{ccc} 2 & 2 & -1\\ 2 & 1 & -1\\ 0 & 2 & -1\\ 0 & 0 & -1 \end{array}\right)\left(\begin{array}{c} 2/3\\ 4/3\\ 8/3 \end{array}\right)=\left(\begin{array}{c} 4/3\\ 0\\ 0\\ -8/3 \end{array}\right).$$

*The index does not provide a separation of the data because $g_{2}=g_{3}=0$. In this case, however, a group $\left\{x_{\left(1\right)},x_{\left(2\right)}\right\}$ dominates $\left\{x_{\left(3\right)},x_{\left(4\right)}\right\}$ in the sense that the difference of averages*

12(x(1)+x(2))−12(x(3)+x(4))=21/2

*is a positive vector.*

*If we set the acceptance ratio to α=1/4, then it is proved in a similar way that the optimal w is $w_{1}=3/4$ and $w_{2}=1$. In this case, c is not unique: 5/2≤c≤7/2. The quantile general index is*

$$\left(\begin{array}{c} g_{1}\\ g_{2}\\ g_{3}\\ g_{4} \end{array}\right)=\left(\begin{array}{ccc} 2 & 2 & -1\\ 2 & 1 & -1\\ 0 & 2 & -1\\ 0 & 0 & -1 \end{array}\right)\left(\begin{array}{c} 3/4\\ 1\\ c \end{array}\right)=\left(\begin{array}{c} 7/2-c\\ 5/2-c\\ 2-c\\ -c \end{array}\right).$$

*Therefore, $g_{1}>0$ and $g_{2},g_{3},g_{4}<0$ as long as 5/2<c<7/2. The separation provides a dominance relation:*

x(1)−13(x(2)+x(3)+x(4))=4/31.

**Example** **3.** *Consider the bivariate data*

x(1)=40,x(2)=24,x(3)=13,x(4)=02

*of sample size 4. Let α=1/2. In a similar manner to the preceding example, the optimal parameters are shown to be $w={\left(1,1\right)}^{⊤}$ and c=4. The quantile general index is*

$$\left(\begin{array}{c} g_{1}\\ g_{2}\\ g_{3}\\ g_{4} \end{array}\right)=\left(\begin{array}{ccc} 4 & 0 & -1\\ 2 & 4 & -1\\ 1 & 3 & -1\\ 0 & 2 & -1 \end{array}\right)\left(\begin{array}{c} 1\\ 1\\ 4 \end{array}\right)=\left(\begin{array}{c} 0\\ 2\\ 0\\ -2 \end{array}\right).$$

*In this case, no separation of the sample into two groups provides a dominance relation. Indeed, all the possible combinations are*

12(x(1)+x(2))−12(x(3)+x(4))=5/2−1/2,12(x(1)+x(3))−12(x(2)+x(4))=3/2−3/2,12(x(1)+x(4))−12(x(2)+x(3))=1/2−5/2,

*which are not positive.*

## 4. Practical Implementation

The quantile general index defined in the preceding section has the following two drawbacks.

- The minimization is not straightforward since *F* is not differentiable.
- The cases with $g_{t}=0$ are not assigned to positive or negative groups.

To overcome these issues, we approximate *F* as

(7) $$\begin{array}{c} F_{\varepsilon }\left(w,c\right)=-\sum_{i=1}^{d}\log w_{i}+\frac{1}{n}\sum_{t=1}^{n}\ell_{\alpha ,\varepsilon }\left(\sum_{i}x_{ti}w_{i}-c\right) \end{array}$$

where ε is a positive constant, and the function $\ell_{\alpha ,\varepsilon }:R\to R$ is defined by

(8) $$\begin{array}{cc} \ell_{\alpha ,\varepsilon }\left(u\right)\; & =\min_{z\in R}\left\{\ell_{\alpha }\left(z\right)+\frac{1}{2\varepsilon }{\left|z-u\right|}^{2}\right\}\\ \; & =\left\{\begin{array}{cc} u/\alpha -\varepsilon /(2\alpha^{2})\; & \mathrm{if}\;u\geq \varepsilon /\alpha ,\\ u^{2}/\left(2\varepsilon \right)\; & \mathrm{if}\;-\varepsilon /(1-\alpha )<u<\varepsilon /\alpha ,\\ -u/\left(1-\alpha \right)-\varepsilon /(2{\left(1-\alpha \right)}^{2})\; & \mathrm{if}\;u\leq -\varepsilon /(1-\alpha ). \end{array}\right. \end{array}$$

**Figure 2:**
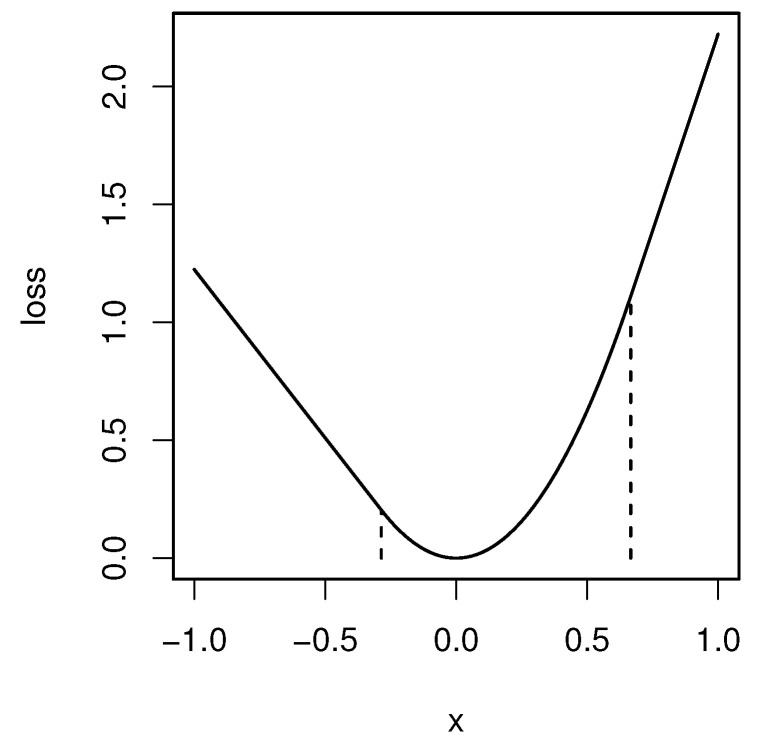
The Moreau envelope of the check-loss function for α=0.3 and ε=0.2. The two vertical lines are u=ε/α and u=−ε/(1−α), respectively.

The function is called the Moreau envelope of $\ell_{\alpha }$. See Figure 2 for the graph of $\ell_{\alpha ,\varepsilon }$. It is shown that $l_{\alpha ,\varepsilon }$ uniformly converges to $\ell_{\alpha }$, as ε→0.

The derivative of $\ell_{\alpha ,\varepsilon }$ is piecewise linear:

$$\begin{array}{cc} \ell_{\alpha ,\varepsilon }^{\prime }\left(u\right)\; & =\left\{\begin{array}{cc} 1/\alpha \; & \mathrm{if}\;u\geq \varepsilon /\alpha ,\\ u/\varepsilon \; & \mathrm{if}\;-\varepsilon /(1-\alpha )<u<\varepsilon /\alpha ,\\ -1/(1-\alpha )\; & \mathrm{if}\;u\leq -\varepsilon /(1-\alpha ). \end{array}\right. \end{array}$$

In particular, $\ell_{\alpha ,\varepsilon }$ is continuously differentiable unlike $\ell_{\alpha }$.

**Definition** **3.** *A general index* $g_{t}=\sum_{i}w_{i}x_{ti}-c$ *is called the quantile general indexwithin tolerance* ε *if* (w,c) *minimizes* $F_{\varepsilon }\left(w,c\right)$ *.*

The gradient of $F_{\varepsilon }$ is

$$\begin{array}{cc} \frac{\partial F_{\varepsilon }}{\partial c}\; & =-\frac{1}{n}\sum_{t=1}^{n}\left(\frac{J_{t}}{\alpha }-\frac{1-J_{t}}{1-\alpha }\right),\\ \frac{\partial F_{\varepsilon }}{\partial w_{i}}\; & =-\frac{1}{w_{i}}+\frac{1}{n}\sum_{t=1}^{n}\left(\frac{J_{t}}{\alpha }-\frac{1-J_{t}}{1-\alpha }\right)x_{ti}, \end{array}$$

where

(9) $$\begin{array}{c} J_{t}=\left\{\begin{array}{cc} 1\; & \mathrm{if}\;g_{t}\geq \varepsilon /\alpha ,\\ \alpha \left(1-\alpha \right)(g_{t}/\varepsilon +1/\left(1-\alpha \right))\; & \mathrm{if}\;-\varepsilon /\left(1-\alpha \right)<g_{t}<\varepsilon /\alpha ,\\ 0\; & \mathrm{if}\;g_{t}\leq -\varepsilon /\left(1-\alpha \right). \end{array}\right. \end{array}$$

These formulas prove the second part of the following theorem. See Appendix A for the proof of the first part.

**Theorem** **4.** *Suppose that there is no hyperplane of $R^{d}$ that contains all $x_{\left(t\right)}$. Then, the objective function $F_{\varepsilon }$ in (8) admits a minimizer (w,c), and the optimal weight vector w is unique. Furthermore, the optimal (w,c) and $J_{t}\in \left[0,1\right]$ defined in (9) satisfy*

(10) $$\begin{array}{c} \frac{1}{n}\sum_{t=1}^{n}J_{t}=\alpha \end{array}$$

*and*

(11) $$\begin{array}{c} \frac{1}{n\alpha }\sum_{t=1}^{n}w_{i}x_{ti}J_{t}-\frac{1}{n(1-\alpha )}\sum_{t=1}^{n}w_{i}x_{ti}\left(1-J_{t}\right)=1. \end{array}$$

The Equations (10) and (11) correspond to (2) and (3) for continuous distributions. The quantity $J_{t}$ is interpreted as the probability of assigning the case $x_{\left(t\right)}$ to the positive group. We call $J_{t}$ *the optimal random decision*. If the general index $g_{t}$ is greater than the threshold ε/α, the case *t* is definitely assigned to the positive group because $J_{t}=1$. Similarly, if the general index is less than −ε/(1−α), it is definitely assigned to the negative group.

For numerical computation, we used a general-purpose optimization solver optim in R [15] with the L-BFGS method.

**Example** **4** (Continuation of Example 2). *Consider four cases*

x(1)=22,x(2)=21,x(3)=02,x(4)=00.

*Let α=1/2 and ε=0.001. The optimal w and c are numerically obtained as $w={\left(0.667,1.332\right)}^{⊤}$ and c=2.666. The quantile general index is $\left(g_{1},g_{2},g_{3},g_{4}\right)=\left(1.333,0.001,-0.001,-2.666\right)$, and the optimal random decision is $\left(J_{1},J_{2},J_{3},J_{4}\right)=\left(1,0.749,0.250,0\right)$, so that the optimal separation will be $\left\{x_{\left(1\right)},x_{\left(2\right)}\right\}$ and $\left\{x_{\left(3\right)},x_{\left(4\right)}\right\}$. This separation happens to satisfy the dominance relation as we have seen in Example 2.*

**Example** **5** (Continuation of Example 3). *Consider four cases*

x(1)=40,x(2)=24,x(3)=13,x(4)=02.

*Let α=1/2 and ε=0.001. The optimal w and c are numerically obtained as $w={\left(1,1\right)}^{⊤}$ and c=4. The quantile general index is $\left(g_{1},g_{2},g_{3},g_{4}\right)=\left(0,2,0,-2\right)$, and the optimal random decision is $\left(J_{1},J_{2},J_{3},J_{4}\right)=\left(0.5,1,0.5,0\right)$. In this case, we cannot decide which of $x_{\left(1\right)}$ and $x_{\left(3\right)}$ has to be assigned to the positive group. This result is consistent with the discussion in Example 3.*

## 5. Application to the SDGs Index

We finally compute the quantile general indices of the SDGs data provided by [1], as introduced in Section 1. According to [1], countries with a fraction of missing values greater than 20% were removed from the data and then the missing values were imputed by regional averages. We applied the quantile general index with the acceptance ratio α=10/163 and tolerance ε=0.001. The result is summarized in Table 2. The optimal weight w is shown in the second column of the table. The threshold was c=178.2. The other columns of Table 2 show the average of each variable in the 10 top countries and the remaining countries, respectively. In contrast to Table 1, we do not observe the reversal relation. Table 3 shows the general index $g_{t}$ and the optimal random decision $J_{t}$ of the 10 top countries.

We must be careful with interpretating the result. In particular, the optimal weights had high variation: the ratio of the largest weight (SDG 12) to the smallest weight (SDG 1) was about 0.49/0.049=10.0, which means that the SDG 1 had only 10% of the impact of the SDG 12 under the quantile general index. This may discourage people or governments contributing to the SDG 1. Our main message in this paper is that there were reversal relations in the SDGs 12 and 13 under the simple sum method, as observed in Table 1, and such a phenomenon can be avoided by the proposed method. Further discussion should be needed for the use of the quantile general index.

As a reviewer suggested, we also computed the Hirsch index [9] (or h-index) of the countries based on the original SDG scores. In the current setting, the h-index is defined as the fixed point of the graph ${\left\{\left(i,s_{i}\right)\right\}}_{i=1}^{17}$, where $s_{i}$’s are the 17 SDG scores in descending order (normalized into the range [0,17]). The 10 top countries based on the h-index are shown in Table 4. The top three were not changed from the original SDG ranking. We also observed the reversal relations in the SDGs 12 and 13 when we adopted the h-index for separation. See [10] for a study of the scaling behavior of the h-index.

## 6. Discussion

We proposed a quantile general index that avoids reversal relations in the separated groups. The weight was defined by the solution of the convex optimization problem (6) or (7) for given data. In Section 5, we applied the proposed method to the SDG data and obtained the 10 top countries based on it. The result actually satisfies the desired properties (10) and (11). A side effect is that the obtained weights sometimes had large variation, which may be controversial.

Various applications of our method are expected. For example, one could construct a regional competitive index (e.g., [16]) based on the quantile general index if it is necessary to select a given number of top regions. The method is also applicable to admission decisions based on entrance examinations in schools or companies, where a fixed fraction of candidates are supposed to pass. Further case studies are needed to support the validity of our approach.

The quantile general index (without approximation) introduced in Section 3 was reduced to a minimization problem of a nondifferentiable objective function. It is theoretically of interest to develop an exact algorithm and also to estimate the accuracy of the practical method developed in Section 4. Another problem is to find an algorithm that decides the separability of the data into two groups without the reversal relations. In Example 3, we enumerated all possible combinations to prove that the data was not separable. However, this algorithm requires a large amount computational time when the sample size is large. Faster algorithms would be welcomed. Finally, the relation between the quantile general index and the h-index is also completely unknown.

## Figures and Tables

**Table 1:** The average values of the SDG scores for the 10 top countries (Finland, Denmark, Sweden, Norway, Austria, Germany, France, Switzerland, Ireland and Estonia) and the remaining 153 countries. The values with the reversal relations are marked by asterisks.

SDGs	Average of the 10 Top Countries	Average of the Remaining Countries
1 (no poverty)	99.6	73.8
2 (zero hunger)	68.5	58.6
3 (good health and well-being)	94.1	68.0
4 (quality education)	98.2	74.9
5 (gender equality)	84.8	60.1
6 (clean water and sanitation)	89.4	66.2
7 (affordable and clean energy)	83.8	64.8
8 (descent work and economic growth)	85.0	66.3
9 (industry, innovation and infrastructure)	91.8	43.2
10 (reduced inequalities)	92.3	59.7
11 (sustainable cities and communities)	92.8	68.8
12 (responsible consumption and production)	60.3 *	85.6 *
13 (climate action)	54.7 *	81.9 *
14 (life below water)	71.4	64.3
15 (life on land)	80.4	64.8
16 (peace, justice and strong institutions)	87.8	65.2
17 (partnerships for the goals)	71.8	58.5

Source: The Sustainable Development Report 2022 [1].

**Table 2:** For the SDGs data, the optimal weight $w_{i}$, the average $x_{i}^{+}$ of each score in the 10 top countries determined from the quantile general index (Cuba, Romania, Finland, Kyrgyz Republic, Ukraine, Chile, Poland, Georgia, Vietnam, Hungary), the average $x_{i}^{-}$ on the remaining 153 countries, and the scaled differences $w_{i}\left(x_{i}^{+}-x_{i}^{-}\right)$ are shown.

SDGs	Weights	Average of the 10 Top Countries	Average of the Remaining Countries	Scaled Difference
i	$w_{i}$	$x_{i}^{+}$	$x_{i}^{-}$	$w_{i}\left(x_{i}^{+}-x_{i}^{-}\right)$
1	0.049	94.9	74.1	1.02
2	0.136	65.8	58.8	0.95
3	0.079	81.3	68.8	0.99
4	0.061	91.4	75.3	0.98
5	0.122	69.2	61.1	0.99
6	0.071	81.3	66.8	1.03
7	0.091	76.0	65.3	0.97
8	0.098	77.4	66.8	1.04
9	0.082	58.4	45.4	1.07
10	0.070	75.5	60.8	1.03
11	0.088	82.0	69.5	1.10
12	0.490	85.9	83.9	0.98
13	0.412	82.4	80.1	0.95
14	0.126	71.5	64.3	0.91
15	0.138	72.6	65.3	1.01
16	0.106	75.4	66.0	1.00
17	0.079	71.2	58.5	1.00

**Table 3:** The 10 top countries based on the quantile general index. The last column shows the original rank based on the SDG scores.

Rank	Country	$g_{t}$	$J_{t}$	Original Rank
1	Cuba	5.09	1.00	40
2	Romania	3.82	1.00	30
3	Finland	3.33	1.00	1
4	Kyrgyz Republic	3.06	1.00	48
5	Ukraine	1.92	1.00	37
6	Chile	1.11	1.00	28
7	Poland	1.03	1.00	12
8	Georgia	0.24	1.00	51
9	Vietnam	0.01	0.68	55
10	Hungary	0.01	0.64	21

**Table 4:** The 10 top countries based on the h-index. The last column shows the original rank based on the SDG scores.

Rank	Country	h-Index	Original Rank
1	Finland	13.47	1
2	Denmark	13.35	2
3	Sweden	13.19	3
4	Germany	13.01	6
5	Romania	12.91	30
6	Norway	12.84	4
7	Estonia	12.77	10
8	Croatia	12.77	23
9	Ireland	12.76	9
10	Portugal	12.64	20

## Data Availability

The SDGs data used in Section 1 and Section 5 is provided in [1].

## Appendix A. Proofs

We give a key lemma for the proof of Theorems 1, 3, and 4. In general, we define

(A1) $$\begin{array}{c} F_{\ell }\left(w,c\right)=\sum_{i=1}^{d}\left(-\log w_{i}\right)+E\left[\ell \left(\sum_{i=1}^{d}w_{i}x_{i}-c\right)\right] \end{array}$$

for $\left(w,c\right)\in R_{+}^{d}\times R$. Here, $\ell :R\to R_{\geq 0}$ is a convex function with properties ℓ(0)=0 and ℓ(u)>0 for u≠0. The check loss function $\ell_{\alpha }$ in Section 2 satisfies these conditions. Under the conditions, we have coercivity

$$\lim_{u\to \pm \infty }\ell \left(u\right)=\infty$$

and subadditivity

ℓ(u+v)≤ℓ(u)+ℓ(v)

for any real *u* and *v*.

We consider the following condition on the nondegeneracy of the distribution P of x. We denote the set of nonnegative numbers by $R_{\geq 0}$.

(C1) $P(\sum_{i}w_{i}x_{i}=c)<1$ for any $\left(w,c\right)\in R_{\geq 0}^{d}\times R$ with w≠0,

This condition holds if P is absolutely continuous with respect to the Lebesgue measure on $R^{d}$, as assumed in Section 2.

Theorem 1 is immediate from the following lemma.

**Lemma** **A1.**
*Suppose that $E[\ell \left(w_{i}x_{i}\right)]<\infty$ for all i=1,…,d and $w_{i}>0$. If the condition (C1) is satisfied, then the function $F_{\ell }$ in (A1) admits a minimizer, and the optimal w is unique. Conversely, if (C1) does not hold, then $F_{\ell }$ is not bounded from below.*

**Proof.** We first show that $F_{\ell }$ is finite everywhere. Indeed, by the subadditivity of *ℓ*, we have

(A2) $$\begin{array}{c} E\left[\ell \left(\sum_{i}w_{i}x_{i}-c\right)\right]\leq \sum_{i}E\left[\ell \left(w_{i}x_{i}\right)\right]+\ell \left(-c\right)<\infty . \end{array}$$

To prove the uniqueness, let $\left(w_{1},c_{1}\right)$ and $\left(w_{2},c_{2}\right)$ be two minimizers of $F_{\ell }$. From the strict convexity of z↦(−logz) and the convexity of *ℓ*, we have

$$F_{\ell }\left(\left(1-\lambda \right)\left(w_{1},c_{1}\right)+\lambda \left(w_{2},c_{2}\right)\right)<\left(1-\lambda \right)F_{\ell }\left(w_{1},c_{1}\right)+\lambda F_{\ell }\left(w_{2},c_{2}\right)$$

if $w_{1}\neq w_{2}$. Thus, we have $w_{1}=w_{2}$, and the uniqueness follows. To prove the existence, we show that the sublevel set

$$\left\{\left(w,c\right)\in R_{+}^{d}\times R|F_{\ell }\left(w,c\right)\leq a\right\}$$

is compact for each a∈R. We define a function $R:R_{\geq 0}^{d}\times R\to R_{\geq 0}$ by

$$R\left(w,c\right)=E[\ell \left(w^{⊤}x-c\right)].$$

Then, *R* is continuous and strictly positive unless (w,c)=(0,0). Indeed, the continuity of *R* is a consequence of Lebesgue’s dominated convergence theorem, and the strict positivity follows from the condition (C1). Let

$$\gamma :=\underset{\sum_{i}w_{i}+\left|c\right|=1}{\inf}R\left(w,c\right)>0.$$

Since *R* is convex, and R(0,0)=0, we have

$$\begin{array}{cc} R(w,c)\; & \geq \gamma \left(\sum_{i}w_{i}+\left|c\right|\right) \end{array}$$

whenever $\sum_{i}w_{i}+\left|c\right|\geq 1$. For any $\left(w,c\right)\in R_{+}^{d}\times R$, we have

$$\begin{array}{cc} F_{\ell }\left(w,c\right)\; & =\sum_{i}\left(-\log w_{i}\right)+R\left(w,c\right)\\ \; & \geq \sum_{i}\left(-\log w_{i}\right)+\gamma \left(\sum_{i}w_{i}+\left|c\right|-1\right)\\ \; & =\sum_{i}\left\{\left(-\log w_{i}\right)+\gamma w_{i}\right\}+\gamma \left|c\right|-\gamma . \end{array}$$

Since the functions $w_{i}\mapsto \left(-\log w_{i}\right)+\gamma w_{i}$ and c↦|c| have compact sublevel sets, the sublevel set of $F_{\ell }$ is also compact. □

In order to prove Theorems 3 and 4, it is enough to replace the distribution P by the empirical distribution $P_{n}=n^{-1}\sum_{t=1}^{n}\delta_{x_{\left(t\right)}}$, where $\delta_{a}$ is the Dirac measure at a point $a\in R^{d}$. In Theorem 3, the uniqueness of *c* when nα is not an integer follows from the observation that the optimal *c* for a fixed w must be $w^{⊤}x_{\left(t\right)}$ for some *t*.
